# Throwing the Baby Out With the Bath Water: Could Widespread Neutering of Companion Dogs Cause Problems at a Population Level?

**DOI:** 10.3389/fvets.2019.00241

**Published:** 2019-07-22

**Authors:** Jessica K. Dawson, Tiffani J. Howell, Matthew B. Ruby, Pauleen C. Bennett

**Affiliations:** ^1^Anthrozoology Research Group, La Trobe University, Bendigo, VIC, Australia; ^2^Department of Psychology and Counselling, School of Psychology and Public Health, La Trobe University, Wodonga, VIC, Australia

**Keywords:** responsible dog ownership, neutering practices, companion dog, dog breeding, anthrozoology

## Abstract

In many countries where companion dogs are popular, owners are strongly encouraged to neuter their dogs. Consequently, millions of dogs are neutered each year. In recent times considerable attention has been paid to the possible effects of such procedures on canine health and welfare. Less scrutinized are the potential ramifications of widespread neutering on the breeding of dogs and their continued success as human companions. This paper summarizes research investigating factors influencing the breeding and rearing of dogs most suited to companionship roles in contemporary, typically high-density, communities, and briefly reviews current breeder practices. It then argues that a fundamental shift to promote inclusion of “proven” companion dogs in the gene pool, as opposed to dogs meeting conformation or working/sporting standards, is required to successfully meet the needs of modern urban dog owners. A new model is proposed, whereby responsible owners and breeders work together to produce dogs most suited for life as human companions.

## Introduction

Dogs are popular companion animals and form relationships with owners that are often deeply important, with many owners perceiving their dogs as family members (1, 2). Out of this high regard was borne the idea of “responsible dog ownership,” a set of behaviors that mark a person as a caring and accountable owner. Relevant behaviors include providing adequate food, water, training, exercise, and veterinary care (3). In many countries, “responsible dog ownership” also involves spaying and castration [e.g., neutering; (4)]. Some places have mandatory neutering laws (5), schemes discounting the procedures (6), and/or campaigns encouraging owners to neuter their dogs (7). These have culminated in neutering becoming a normative practice in countries such as the United States of America (USA), Australia, and New Zealand (8, 9).

As neutering procedures have become more common, the effects of neutering on canine health and behavior, and its use as a population management tool, have been examined (10, 11). Less attention has been paid to the possible effect of widespread neutering on the breeding of dogs and their success as human companions. This paper considers this issue.

## History and Current Cultural Understanding of Neutering

At the beginning of the twentieth century, most dogs never saw a veterinarian (12, 13) and neutering was a relatively unknown procedure (14). This began to change in the early 1970s in the USA, when the size of the stray population attracted public attention and resulted in the opening of the first subsidized neutering clinic in 1971 (15).

Today, neutering is one of the most common surgeries performed on companion animals in many countries (16). In some cultures, having one's dog neutered is considered an essential part of being a “responsible owner,” and rates range from ~60–80% (9, 17). Prevalence varies tremendously, however, as a function of differing societal attitudes (18). In several European nations, such as Norway, neutering healthy dogs, perceived as unnecessary mutilation, is closely monitored or even prohibited (19). In such countries, neutering rates range from 43% to as low as 1% (20). Differences in neutering prevalence also exist within countries Diesel et al. (21), depending on factors such as socio-economic status (18, 22), owner gender (23), dog sex (21, 24), and owner perceptions regarding the practice and its implications (8, 23).

Downes et al. (25) identified several perceptions that act as barriers to neutering, including the financial costs, adequacy of alternative methods for preventing reproduction (i.e., high fences), and potential negative effects on the dog's health and welfare (25). Perceptions linked to pro-neutering attitudes included perceived positive health effects and a reduction in unwanted behaviors among neutered dogs. As companion dogs have moved from rural yards into urban bedrooms, expectations regarding behaviors once considered normal have changed.

## The Ideal Companion Dog: What do we Want and how can we get it?

Ensuring that one's chosen dog is compatible with one's lifestyle is an important step in the acquisition process. For this reason, potential owners are often encouraged to investigate what kind of dog (e.g., breed, sex, age) would be most suitable (26). Research has shown that most people judge personality and behavior as the most important factors to consider when acquiring a dog (27). King et al. (28) found that favored traits for Australian owners included the degree to which a dog is affectionate, loyal, friendly, obedient, easily contained, and safe with children. Similar results were obtained when the same survey was administered in Italy (29).

While puppy temperament tests are notoriously unreliable in terms of predicting adult dog behaviors (30), evidence suggests that desirable canine characteristics, such as those identified by King et al. (28), are largely within the realm of human control. Canine personality refers to a constellation of psychological attributes that underpin consistent patterns of behavior and that are resistant to change (31). In most organisms, including dogs, personality is relatively fixed by adulthood, having developed due to interactions between the animal's genetically determined temperament and what they experienced during development (32). Both of these components reflect choices made by those who breed dogs.

Scott and Fuller (33) pioneered research on genetic contributions to canine personality through extensive experimentation with dogs of known heritage. Their work demonstrated the substantial influence genetics has on the development of traits such as emotional reactivity, trainability, problem solving, and aggressiveness. These findings have been explored more recently in large community-based cohorts and similar results have been obtained, particularly concerning traits such as shyness-boldness (34), affability (35), and aggressive tendencies (36). There has also been research to suggest that some traits may be passed onto offspring epigenetically as a result of parental experiences (37).

The second major influence on canine personality is each dog's environmental experience, particularly in the first few months of life (38). This is due to the period of heightened sensitivity to experiences that juvenile dogs undergo, which begins at 2–3 weeks of age and lasts until the puppy is ~12–14 weeks old (33). As experiences in this time form the foundation for adult personality, it is strongly recommended that puppies be exposed, in a positive way, to the full range of experiences they are likely to encounter as adults (39, 40).

Ensuring that puppies are reared in a place plentiful in diverse experiences is an invaluable step to setting them up for the life they are likely to lead (41). Puppies who are reared inside a home, at least for a period of time, rather than in a kennel, have been found to perform better on tests that measure social attraction and cooperativity, and they display lower levels of aggression and apprehensive behavior (42, 43). The degree of human handling experienced has also been shown to be influential in the development of traits such as confidence, calmness, and stress resilience, with more experience leading to better outcomes (44–46).

## Current Companion dog Breeding Practices

The demonstrated importance of genetics and early environment in determining behavioral predispositions makes it imperative to consider where companion dogs come from. Prior to the widespread introduction of neutering practices, dogs often bred indiscriminately, and people typically obtained their dogs for free from neighbors whose bitch had produced a litter (47). While this was problematic in terms of creating dog overpopulation, it meant that most of the dogs who produced offspring were well suited to the demands of the lives they were expected to lead. Those who weren't well-suited were disposed of. Today, strong demand for companion dogs, coupled with rapid urbanization, increased concern regarding the welfare of animals, particularly companion dogs, and high neutering rates, has resulted in a multimillion-dollar industry involving the selective breeding and selling of puppies (48). Widespread neutering means that humans intentionally control nearly all dog breeding in developed countries.

Only limited evidence exists regarding how dogs are bred, particularly about how breeding choices are made and how puppies are reared (49). One prominent group of breeders are those who breed purebred dogs as a hobby, often secondary to their participation in competitions with their dogs, such as conformation showing or agility. These breeders are usually members of interest groups such as kennel clubs and, in line with the regulations of these clubs, are expected to adhere closely to written breed standards, documents which stipulate the ideal characteristics of the breed, when selecting dogs for breeding (50). Breeders in this group breed ostensibly only to “improve the breed” as defined by this standard, rather than to meet the demands of the companion dog market (51, 52). This has led to exaggerated phenotypes associated with serious health problems (53). In addition, it may lead to production of dogs with behavioral characteristics more suited to their traditional roles as herders, retrievers or guard dogs, than they are to a life where they are expected to be friendly, obedient, affectionate, easily contained, and safe with children. While many hobby breeders do rear their puppies in their homes and sell most of them as companion animals, an additional concern is an emerging trend for “responsible” breeders in this group to neuter puppies prior to sale. This has implications in terms of the health and development of the puppies (54), but also means that breeders must select their breeding dogs at a very young age, prior to seeing how they develop as adults. It therefore serves to potentially remove many of the very best companion dogs from the gene pool.

Another broad group of breeders are commercial breeders, who breed for the primary purpose of making a profit. Unlike hobby breeders, commercial breeders often specifically produce dogs for the companion market (48). For their enterprise to be viable, these breeders usually operate on a larger scale than hobby breeders. One might question whether large-scale breeding facilities are able to provide adequate environmental stimulation and socialization practices for puppies (55). To some extent this depends on the dog-to-human ratio and the actual facilities available. Breeders who operate “puppy farms,” are frequently condemned due to overcrowding, poor sanitation and other issues (55). However, even the best commercial establishments may be less able to provide the simulated “home environment” to which puppies destined for companionship life should be exposed. Bennett and Rohlf (56) and McMillan et al. (57) identified significantly more behavioral issues, such as fear, anxiety, and aggression, in dogs coming from pet shops, many of which source their puppies from commercial breeders.

The third, and likely the largest, companion dog breeder group is the general public (58, 59). Importantly, however, not all companion dog owners are equally likely to produce puppies. As described previously, in many developed countries, neutering companion dogs is considered an important aspect of responsible ownership. Hence, the very best companion dogs in the general community, those owned by responsible citizens who choose their dogs carefully and ensure they are reared correctly, are almost certainly those most likely to be neutered. Conversely, it is those companion dog owners who fail to perform the “responsible” behavior of neutering their dog who are perhaps most likely to breed. These “breeders” may also choose not to perform other “responsible” behaviors, such as selecting their dog carefully, testing it for genetic disorders, or evaluating the dog's suitability as a companion prior to allowing it to reproduce. In other words, they may not thoroughly consider the genetic and environmental factors known to be critical to optimal puppy development.

Irresponsibly bred puppies are at high risk of being relinquished. In fact, millions of puppies enter shelter and rescue systems each year (60). Very little information exists regarding the puppy rearing practices undertaken by shelters and rescues, though these are likely extremely variable and dependent on available resources. Such organizations have no control over which dogs are bred and often lack access to information about puppies' parentage. It is also likely that, in many cases, they may not be equipped with the knowledge, time, or resources to rear puppies in an environment that provides extensive or appropriate opportunities for socialization. Owners of dogs acquired from a shelter or rescue group have previously reported higher levels of undesirable traits, such as fearfulness and unfriendliness/aggression, though the likelihood is lower if the dog is acquired as a puppy (56, 61).

The main breeder groups identified above are by no means exhaustive, mutually exclusive or distinct. Many people who would describe themselves as hobby breeders do not breed purebred dogs but focus instead on specific breed crosses they believe are excellent human companions. Some commercial breeders do not focus solely on profit, but report paying careful attention to the welfare and suitability of their dogs (62, 63). Breeders in all groups routinely claim that they rear companion puppies in environments that set them up for success, and also that they ensure breeding dogs possess traits desired by companion dog owners. Whether these assertions are correct or not is difficult to assess, a situation that leaves both breeding dogs and the general public vulnerable to exploitation. For dog owners, the risks may be exacerbated by increased urbanization, which increases social demands on dogs but also means that many owners lack prior exposure to animals, a situation vastly different from just a few decades ago, when most community members were familiar with a broad range of domesticated animals. Current breeder practices, supported in part by the higher prices and increased demand for puppies associated with widespread neutering, may mean that many dogs end up in environments to which they are poorly suited, and that many dog owners end up with a dog which is poorly suited to their lifestyle and expertise.

## Bringing it all Together: An Alternative Approach to Neutering

To increase the chances of harmonious relationships forming between modern owners and their companion dogs, we believe three changes should be made to current breeding practices. First, it is imperative that breeding choices and puppy rearing processes, such as whelping and socialization procedures, are informed by empirically derived knowledge of best practice. We believe that all breeders should be educated to understand the critical roles they play in providing dogs suited for the companion market, both through the adult dogs they choose to breed from and the early experiences they provide to puppies. To facilitate putting this education into practice, we believe that breeding choices and puppy rearing processes should be clearly documented and archived, with this information being provided to purchasers alongside other required documentation such as veterinary records and microchip information.

Second, we advocate that all dogs should be independently tested for suitability before being bred—much as breeders now advertise that their puppies' parents are successful show dogs, or that they are free from known genetic disorders, so they should be encouraged to advertise that independent testing has shown their breeding dogs to be well-suited behaviourally to life as human companions. We anticipate that responsible breeders would be willing to pay for this independent certification, much as they presently pay for genetic tests, eye screening and tests for hip dysplasia. Several behavioral tests exist to measure specific traits, such as the Socially Acceptable Behavior test (64), which measures aggression, or the Dog Mentality Assessment test (65), which examines levels of playfulness, curiosity, aggression, sociability, and chase-proneness. In the USA, the Canine Good Citizen program, administered by the American Kennel Club, takes <30 min to administer and is designed to identify dogs that meet ten objectives consistent with being a good companion dog. Any one of these tests could be used as a basis for developing an assessment suited to breeding dogs—dogs that are not themselves good companions are less likely to produce puppies able to excel at this role.

Third, a collaborative approach should be promoted between breeders and companion dog owners, whereby owners are invited to play a critical role in the breeding process. Whilst breeders may possess the skills necessary to breed dogs and rear puppies successfully, companion dog owners are often in the best position to enable dogs to “prove” themselves as suitable breeding dogs through observing their dog's responses to a range of relevant experiences.

These changes are achievable only if the current approach to neutering is altered. At present, widespread neutering of companion dogs undermines the production of suitable dogs by excluding thousands of ideal companions from the gene pool. These include dogs produced by “responsible” breeders, who are neutered before being sold as puppies or who come with a contract stipulating they be neutered by a certain age. They also include dogs owned by “responsible” owners, who ensure that their dogs participate in appropriate training and socialization activities and that they are fully integrated into the household, and who also “do the right thing” by ensuring that their dogs are neutered. While we agree that dogs with unknown histories should continue to be routinely neutered, as should dogs from parents known not to be suitable as companion dogs, we advocate for a more nuanced approach to neutering, in which puppies from dogs carefully chosen for their companionships traits and placed in homes with owners who agree to prevent breeding until their dog can be thoroughly evaluated, should have the opportunity to remain intact.

If, as adults, these dogs demonstrate suitability as breeding dogs, they could be temporarily returned to the breeder for breeding purposes. Alternatively, if the breeder does not have the resources and time to rear the puppies inside their home, select owners could whelp their bitch under close guidance of an experienced breeder. This model could provide puppies with an optimal early environment, closely approximating the one in which they will be expected to live as adults, and it would allow breeders access to a wider pool of dogs for breeding. It would also prevent the current situation, in which breeders must select which dogs to retain for breeding at a very young age, before adult traits are apparent. This could strengthen the gene pool and reduce inbreeding.

Such a collaborative approach to breeding, involving breeders working closely with some owners, is not entirely novel, with an internet search revealing similar approaches currently being used by some organizations breeding dogs to work as guide dogs or as assistance animals, as well as a small number of companion dog breeders. However, such practices are neither common nor well-documented, so it is unknown whether those individuals we located implement all of the above recommendations and/or how they do so. Furthermore, we could find no evidence of any formal evaluation of these programs, so it is not known to what extent they are successful.

To summarize, we propose a systemic restructure of the companion dog breeding industry that incorporates increased transparency regarding breeding choices and puppy rearing practices, temperamental evaluation of breeding dogs, and a collaborative relationship between breeders and owners that allows for more suitable dogs to remain within the gene pool. We acknowledge that this model is not without risk. Breeders and owners may disagree over many things, such as whether a particular dog is suitable for breeding and who owns any puppies produced. The potential for indiscriminate breeding also exists, and this could contribute to further overpopulation and increase pressure on shelters and rescue groups, although we think it is less risky at a population level than the current practice of removing the very best companion dogs from the breeding pool. Adverse situations could be prevented by careful recruitment of owners who are suitable, well-informed, and sufficiently supported, alongside clear contractual agreements that protect both the humans and dogs involved. Over the long term, a more considered approach to the breeding of companion dogs would help lessen the gap between owner expectations and the dogs available to them. However, this is only possible if attitudes toward neutering are addressed and “responsible ownership” is broadened to include a dynamic partnership between owners and breeders to produce dogs most suited for life as companions.

## Author Contributions

PB and JD conceived of the proposed idea. The manuscript was written and revised by JD with the support and contributions of TH, MR, and PB.

### Conflict of Interest Statement

The authors declare that the research was conducted in the absence of any commercial or financial relationships that could be construed as a potential conflict of interest.
